# Printed Electrode for Measuring Phosphate in Environmental Water

**DOI:** 10.1021/acsomega.1c00132

**Published:** 2021-04-22

**Authors:** Alisha Prasad, Sushant P. Sahu, Sara Karoline Figueiredo Stofela, Ardalan Chaichi, Syed Mohammad Abid Hasan, Wokil Bam, Kanchan Maiti, Kevin M. McPeak, Gang Logan Liu, Manas Ranjan Gartia

**Affiliations:** † Department of Mechanical and Industrial Engineering, Louisiana State University, Baton Rouge, Louisiana 70803, United States; ‡ Department of Chemical Engineering, Louisiana State University, Baton Rouge, Louisiana 70803, United States; § Department of Oceanography and Coastal Sciences, Louisiana State University, Baton Rouge, Louisiana 70803, United States; ∥ Department of Electrical and Computer Engineering, University of Illinois, Urbana-Champaign, Illinois 61801, United States

## Abstract

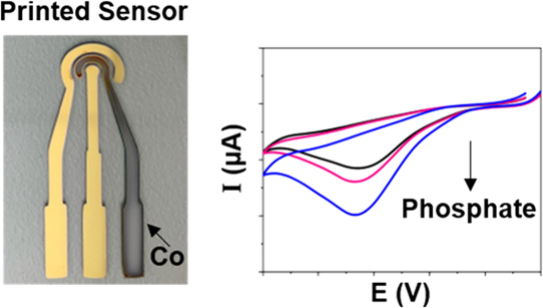

Phosphate is a major nonpoint source pollutant in both the Louisiana local streams as well as in the Gulf of Mexico coastal waters. Phosphates from agricultural run-off have contributed to the eutrophication of global surface waters. Phosphate environmental dissemination and eutrophication problems are not yet well understood. Thus, this study aimed to monitor phosphate in the local watershed to help identify potential hot spots in the local community (Mississippi River, Louisiana) that may contribute to nutrient loading downstream (in the Gulf of Mexico). An electrochemical method using a physical vapor deposited cobalt microelectrode was utilized for phosphate detection using cyclic voltammetry and amperometry. The testing results were utilized to evaluate the phosphate distribution in river water and characterize the performance of the microsensor. Various characterizations, including the limit of detection, sensitivity, and reliability, were conducted by measuring the effect of interferences, including dissolved oxygen, pH, and common ions. The electrochemical sensor performance was validated by comparing the results with the standard colorimetry phosphate detection method. X-ray photoelectron spectroscopy (XPS) measurements were performed to understand the phosphate sensing mechanism on the cobalt electrode. This proof-of-concept sensor chip could be utilized for on-field monitoring using a portable, hand-held potentiostat.

## 1 Introduction

Population growth, environmentally unsound agricultural practices, and increased industrialization are the prime drivers in the accumulation of excessive nutrients (nitrate, phosphate, ammonia) in water bodies, leading to eutrophication.^1,2^ This eutrophication of water bodies results in algal blooms and low oxygen levels, referred to as hypoxic water, that can kill fish and seagrass, impacting the viable habitat available to aquatic life. It can also lead to harmful algal blooms (HABs) that affect the biological lifecycle, human health, and natural beauty.^3−5^ Total phosphorous (P) in the concentration range of 0.02–0.1 mgL^–1^ is considered the amount defining the onset of eutrophication in water bodies where P is the limiting nutrient.^6−8^ Even in bodies of water where P is not the limiting nutrient, the N/P ratio can determine the phytoplankton communities and the development of HABs.^9−11^ For these reasons, The U.S. Environmental Protection Agency (EPA) has set the maximum allowable contaminant level (MCL) for total phosphorus to 0.05 to 0.5 mg L^–1^.^12^ However, phosphorus measurement at such low concentrations in natural water on a routine basis to check for eutrophication is challenging. Currently, the only EPA-approved method for phosphorus detection is the spectrophotometric-based Method 365.5.^13^ In this approach, dissolved and/or suspended orthophosphates react with a cocktail of reagents such as ammonium molybdate, antimony potassium tartarate, and ascorbic acid in an acidic medium to produce a blue complex, the optical absorbance of which is measured spectrophotometrically.^14^ Although this method is sensitive to the detection of low concentrations of phosphate, its disadvantages include decay in color, refractive index errors, and turbidity interferences.^15,16^ Besides, since this method relies on a batch process, it is not suitable for field implementation or online monitoring systems. However, some of these disadvantages have been addressed recently by quickly measuring the final sample analytes,^17^ using high-quality quartz cuvettes,^18^ and filtering samples to measure the soluble reactive phosphorus (SRP) and blank corrections.^19^

The primary advantage of using *in situ* sensors in the molybdate-spectroscopy method is that it allows for obtaining data in real time, monitoring at the higher temporal resolution, and reducing the cost. Researchers have also automated the classic colorimetric molybdenum-blue batch method of phosphate sensing, developing a Lab-On-Chip (LOC) Phosphate Analyzer using microfluidics^20^ and optofluidic systems^21^ for the detection of phosphate in oceans. However, these colorimetric-based methods experience decay in color over time; since a bulky and power-intensive system is utilized, continuous monitoring with high throughput is challenging. Despite these disadvantages, commercial systems such as Sea-Bird Scientific’s HydroCycle-PO4 Phosphate Sensor (OTT HydroMet, USA) and the A1000 Phosphate sensor (Dartmouth Ocean Technologies Inc., USA) are available to perform *in situ* measurements of phosphate at sea.

Recently, alternative approaches such as electrochemical phosphate detection have received much attention due to their potential for miniaturization. Several studies have reported enzyme-based electrodes,^22,23^ carbon paste electrodes,^24,25^ and glassy carbon electrodes^26^ for detecting phosphate. The amperometric or voltammetric signals obtained are based on the oxidation–reduction complex formed due to redox reactions of cobalt. Past research has reported that cobalt-based sensors have achieved a limit of detection (LOD) in the micromoles per liter range. Furthermore, phosphate sensors utilizing enzyme-based electrodes experience enzyme inactivity when used for a long period of time and potential issues with reproducibility in fabricating the enzyme immobilized electrodes with a proper enzyme orientation to sense phosphate analyte. On the other hand, the sensitivity of carbon-based electrodes is limited due to high electrochemical noise. To address this limitation, several studies have researched the use of ion selective metal electrodes such as cobalt wire,^27^ cobalt microelectrodes,^28,29^ cobalt screen printed electrodes,^30,31^ and lead wire^32^ for phosphate detection. Among these, cobalt is an attractive choice as it produces a high signal-to-noise ratio and can be miniaturized through physical vapor deposition (PVD) methods, although it can experience oxidation upon repetitive use. As the use of lead as a working electrode is limited due to its toxicity issues, here, we used cobalt as the working electrode. Since our goal is to develop field-applicable single-use sensor chips, metal oxidation can be tolerated.

Although significant progress has been made in the development of *in situ* phosphate sensors,^31,33−36^ the transport of phosphate ions in environmental water leading to eutrophication is not yet well understood. Because the increase in phosphorus contamination is reaching critical levels in water basins around the globe, there is an urgent need to understand the transport of nutrients and devise a strategy to mitigate these pollutants at the local watershed. To actively manage the nutrients leading to environmentally compromised water bodies, we need to first measure them. Given this motivation, the aim of this research was to develop *in situ* sensors for the electrochemical detection of phosphate in environmental water.

This work is one of the first to monitor phosphate in the local watershed to help recognize possible ″hot spots″ in the local community that may be contributing to nutrient stacking downstream. First, electrochemical methods using cyclic voltammetry and amperometry were utilized to detect phosphate in water samples. We used a cobalt-based miniaturized working electrode for the continuous detection of phosphate (quasi-transient) in water samples over a one month period. We evaluated the performance of the sensor by rigorously establishing the limit of detection, the effect of additional parameters such as pH and dissolved oxygen, and the presence of interfering ions in the water samples. Furthermore, the performance of the sensor was compared with the standard color-based phosphate detection method using spectrophotometry.

## 2 Results and Discussion

### 2.1 Electrochemical Phosphate Sensing and Evaluation of Dynamic Range

**Figure 1:**
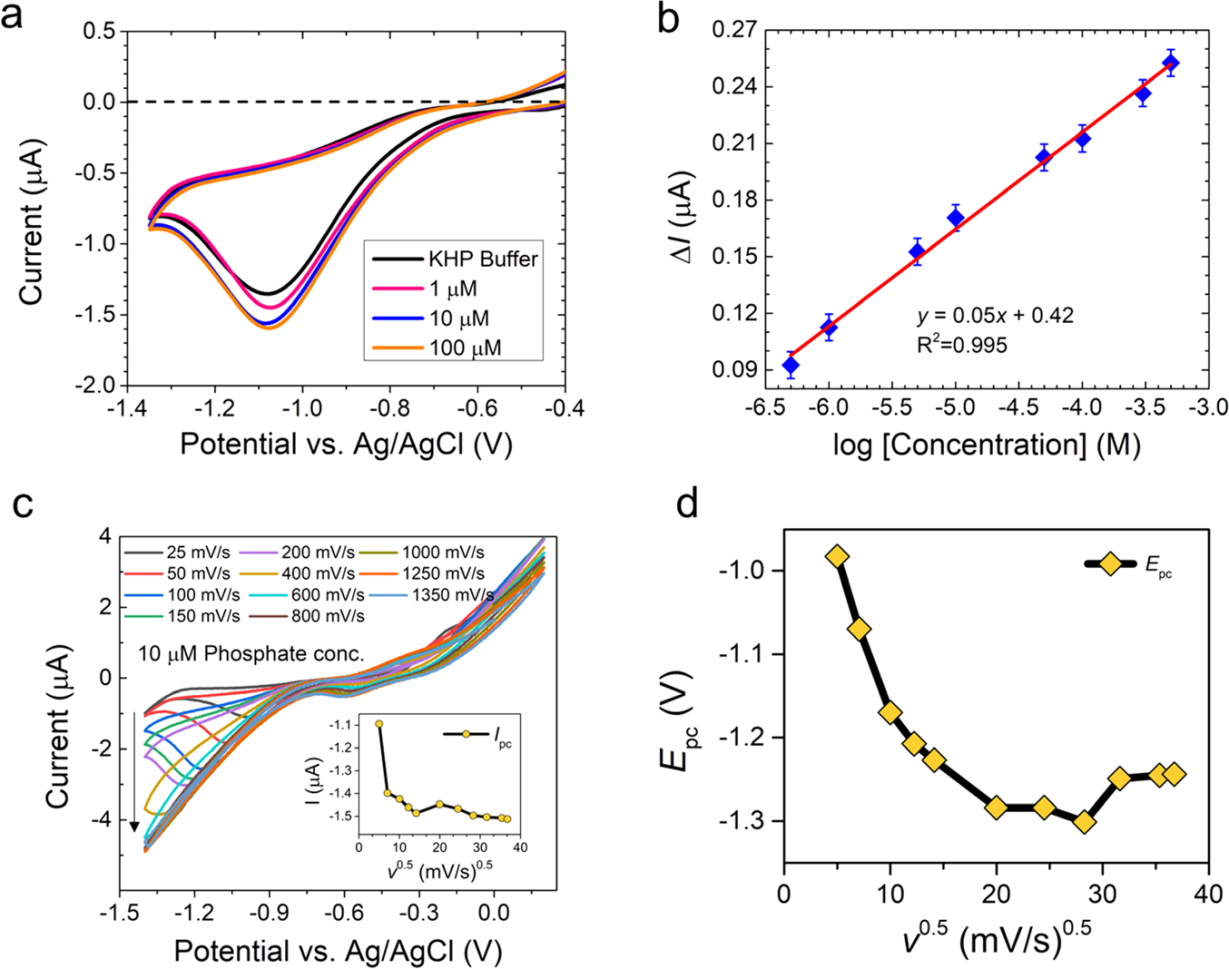
Cyclic voltammetry (CV) results using the phosphate sensor. (a) Cyclic voltammograms with a cobalt wire as the working electrode, Ag/AgCl as the reference electrode, and Pt wire as the counter electrode in a 25 mM KHP buffer and 1 mM KCl with KH_2_PO_4_ in a concentration range of 1 to 100 μM at a scan rate of 50 mV/s. (b) Phosphate sensor calibration curve showing a linear range of detection from 1 to 100 μM with bulk cobalt wire. The current signal obtained from the buffer was subtracted from all sample peak currents. (c) Effect of scan rate from 25 to 1350 mV/s on the performance of the phosphate sensor at a constant phosphate concentration (∼10^–6^ M). Inset shows *I* vs scan rate^0.5^. (d) *E* vs scan rate^0.5^.

According to the Henderson–Hasselbalch equation, the dissociation of KH_2_PO_4_ follows
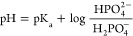
1where the potassium dihydrogen phosphate (KH_2_PO_4_) ionizes under equilibrium conditions at pH ∼ 4 into two species: dihydrogen phosphate (H_2_PO_4_^–^), a **weak acid**, and hydrogen phosphate (HPO_4_^2–^), its **conjugate base**. At pH ∼ 4 and pK_a_ = 7.198, [HPO_4_^2–^/H_2_PO_4_^–^] = 3 × 10^–3^, meaning that the species we are detecting using the electrochemical measurement will predominantly be H_2_PO_4_^–^ (the eq 4 complex in Supplementary Information Section S1 represents the same result). As phosphate sensing in the biological environment is challenging, this study used the electrochemical method for signal transduction and direct conversion of the biological analyte to a detectable electronic signal. Electrochemical responses were recorded by performing CV measurements involving redox reactions (oxidation and reduction) occurring on the working electrode surface. Figure 1a shows the CV curve obtained at a scan rate of 50 mV/s for the cobalt sensor in a 25 mM potassium hydrogen phthalate (KHP) buffer with a pH ∼ 4. Eight concentrations of standard phosphate solution (orthophosphate) were prepared in the range of 10^–7^ to 10^–4^ M KH_2_PO_4_ for calibration. H_2_PO_4_^–^ was the dominant species at this pH range. The CV curve was obtained by scanning the potential from −1.4 to 0.4 V vs Ag/AgCl. The current–potential (*I*–*V*) curve showed anodic current at 0.4 V due to the oxidation of Co^0^ to Co^2+^. Furthermore, due to the reduction of Co^2+^ to Co^0^, a cathodic current wave was generated with a reduction peak around −1.0 V, a result that agrees with the values found in the literature.^31^

Figure 1b shows the calibration curve for phosphate ions in the range of 10^–7^ to 10^–4^ M KH_2_PO_4_ calculated from the CV measurement data. It shows a linear relationship over the dynamic range with a slope of 0.05 μA/decade. The dynamic range found here is at least two orders of magnitude broader than previously reported values. (The previously reported dynamic range was from 10^–5^ to 10^–2^ M.^27,28,31,37,38^) All of our electrochemical experiments were conducted at 50 mV/s as the CV curves were stable at this scan rate with a good reduction peak at approximately −1.05 V. The limit of detection of the cobalt-wire sensor was estimated to be ∼10^–7^ M using the relationship 3σ_b_, where σ_b_ is the standard deviation of the buffer solution containing no phosphate. Experiments were conducted at least three times at each concentration to determine the standard deviation. The magnitude of the current response of the buffer was subtracted from all sample peak current responses to plot the calibration curve. The *R*^2^ coefficient of the linear fit is 0.996.

The quasi-reversible electron transfer process in the resulting cyclic voltammograms can be represented by the Randles–Sevcik equation below:
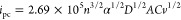
2where *i*_pc_ is the peak current, *n* is the number of electrons transferred, *A* is the area of the electrode, α is the electron transfer coefficient, *D* is the diffusion coefficient, *v* is the scan rate, and *C* is the concentration of the bulk solution. Figure 1c shows the CV curve at different scan rates from 25 to 1350 mV/s, and the inset plot of the *I* vs scan rate^0.5^ shows that at high scan rates (where *v*^0.5^ is small), the cobalt redox couple exhibits an irreversible behavior. Since the couple exhibits an expected reversible behavior up to a scan rate of 200 mV/s, all CV experiments were conducted at 50 mV/s.

The deviation of the peak potential *E* vs scan rate^0.5^ is shown in Figure 1d.

The kinetics of the chemical reaction and the diffusion of orthophosphate ions from the solution to the electrodes were further explored using chronoamperometry (CA). Supplementary Information Figure S2 shows the current–time profile for the phosphate ions at various concentrations ranging from 10^–7^ to 10^–4^ M KH_2_PO_4_, obtained by setting the working electrode at 0.25 V at a step time of 40 s against an Ag/AgCl electrode. The plot in Figure S2a shows an increase in current with an increase in analyte concentration, as demonstrated by the step responses. The plot in Figure S2b of the calibration curve for the orthophosphate ions calculated from the CA measurements data shows a linear relationship over the dynamic range with a slope of 0.13 μA/decade.

**Figure 2:**
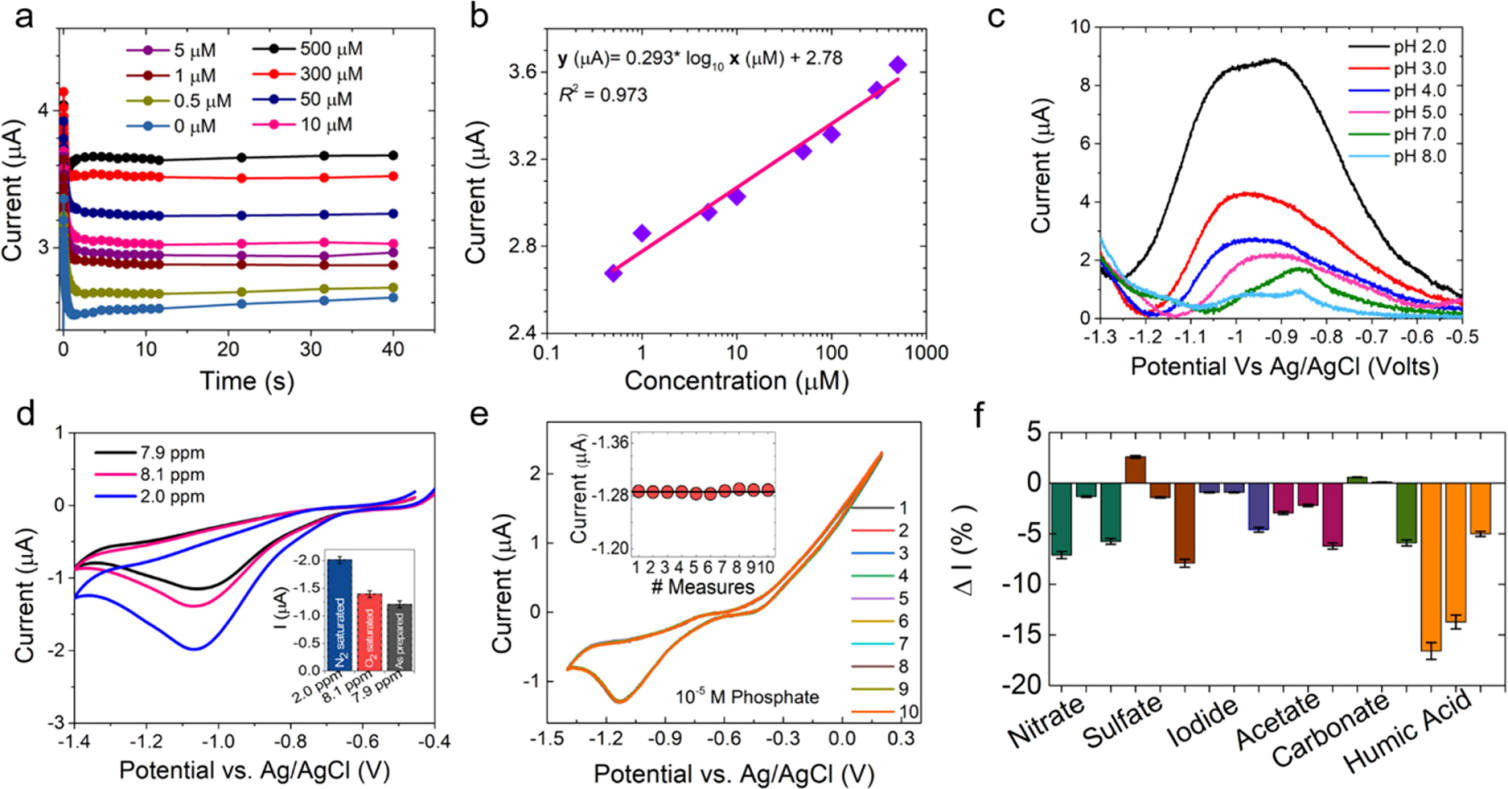
Current–time response profile of the phosphate sensor. (a) Chronoamperograms showing the phosphate sensor response at different phosphate concentrations. (b) Phosphate sensor calibration curve showing a linear range of detection from 5 × 10^–7^ to 5 × 10^–4^ M with bulk cobalt wire. The current signal obtained from the buffer was subtracted from all sample peak currents. The intra-assay coefficient of the variation percentage (CV%) (*n* = 8) was calculated to be 4.4%. (c) Evaluation of cobalt sensor response in the pH range of 2 to 8. Square wave pulsed voltammograms show the characteristic cathodic current of 10^–4^ M aqueous KH_2_PO_4_ at various pH ranges from 2 to 8 in a 25 mM KHP buffer and 1 mM KCl at a scan rate of 50 mV/s. (d) Evaluation of the effect of dissolved oxygen on the cobalt phosphate sensor. Inset: Difference in current response measured at the cathodic peak potential (−1.05 V). (e) Evaluation of the electrochemical stability of the cobalt-based phosphate sensor at a fixed phosphate concentration (∼10^–5^ M). Inset: Variation in the current response measured at the cathodic peak potential (−1.05 V) for 10 measurements. (f) Evaluation of the cobalt sensor response in the presence of various interfering cations. The bar graph shows the change in the current response measured at the cathodic peak potential (−1.05 V). Left to right: nitrate (0.05, 0.5, and 5 mM); sulfate (0.09, 0.9, and 9 mM); iodide (0.05, 0.5, and 5 mM); acetate (0.05, 0.5, and 5 mM); carbonate (0.05, 0.5, and 5 mM); and humic acid (5, 0.5, and 0.25 mM).

To evaluate the diffusion of the species on the cobalt electrode surface, we plotted the current–time profile for the phosphate ions at individual concentrations and extrapolated the diffusion coefficient using the Cottrell^39^ equation:
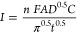
3where *I* is the current (A), *n* (≈4) is the number of electrons reduced or oxidized, *F* is the Faraday constant (96,485 C/mol), *A* is the area of the electrode (1.85 cm^2^), *D* is the diffusion coefficient (cm^2^/s), *C* is the concentration of the reducible analyte (mol/cm^3^), and *t* is the time (s). Figure 2a shows the plot of *I* vs *t* at different concentrations. The diffusion coefficient at 5 μM (∼5 × 10^–9^ mol/cm^3^) phosphate concentration calculated using eq 3 at *t* = 10 s and *I* = 2.94 μA was found to be 2.1 × 10^–5^ cm^2^/s. The calculated value of the diffusion coefficient is in good agreement with the literature value of the diffusion coefficient of PO_4_^3–^ in water (which is ∼1 × 10^–5^ cm^2^/s^40^), while in the flocs, it is 7.6 × 10^–5^ m^2^ d^–1^.^41^ The plot in Figure 2b shows the calibration curve for the orthophosphate ions obtained from the measurement data in Figure 2a**.**

### 2.2 Effect of Environmental Interferences and Electrochemical Stability

To check the cobalt sensor’s selectivity in detecting phosphate ions and evaluating the influence of other interfering factors on the performance of the phosphate sensor, the following tests were performed.

#### 2.2.1 Effect of pH on the Phosphate Sensor

The thermodynamic equilibrium state of the metal–electrolyte system at varying pHs in an electrochemical system can be investigated using the Pourbaix diagram. Supplementary Information Figure S3 shows the Pourbaix diagram of cobalt. The cathodic (orange bullets) and anodic (blue bullets) peak potential obtained from the CV curve at pH = 2 to 11 was plotted on the Pourbaix diagram. The corresponding square wave pulsed voltammograms showing the characteristic cathodic current curves at different pH conditions are also presented in Figure 2c. The corresponding CV curves are shown in Supplementary Information Figure S5**.** The plots from the Pourbaix diagram and the CV curves suggest that the sensor response is pH-dependent. The availability of nutrient phosphate is significantly affected by the pH condition of the water.^42,43^ The ideal pH condition for most algae growth falls slightly within the alkaline pH range of 8.2 to 8.7.^44^ However, certain algae species, for example, *Spirodela polyrrhiza,* grow at a slightly acidic pH between 6 and 6.5.^45^ As a result, for this project, we have recorded the response of the electrochemical phosphate sensor over a broad pH range.

#### 2.2.2 Effect of Dissolved Oxygen on the Phosphate Sensor

Dissolved oxygen (DO) is known to steadily deplete in areas rich in microalgal bacterial flocs.^46^ To closely mimic the environmental settings and confirm the stability of the cobalt-based phosphate sensor under such conditions, we evaluated the effect of DO on the electrode surface. Figure 2d shows three CV curves under the following conditions: as-prepared, air/O_2_-saturated (increase in DO), and N_2_-purged (decrease in DO) at a 10^–2^ M constant aqueous phosphate concentration. The concentration of DO, measured using a commercially available oxygen electrode (YSI, USA), was found to be 7.94 ppm for as-prepared, 8.08 ppm for air/O_2_-saturated, and 2.02 ppm for N_2_-saturated phosphate solutions. The response of the phosphate sensor was influenced by the DO concentration as assisted by the cathodic reduction peak observed at around −1.05 V due to oxygen reduction reaction in eq 4 below:
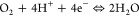
4

The current response changed from −1.2 to −2.0 μA (∼66% change) when the DO changed from 2 to 8 ppm (inset Figure 2d). This result suggests that DO needs to be measured along with the sensor response for any practical application as it will interfere with an analyte.

#### 2.2.3 Electrochemical Stability

In addition, the electrochemical stability of the cobalt sensor was evaluated by recording the CV 10 times at the same 10^–5^ M phosphate concentration, as shown in Figure 2e. The inset plot of the current vs the number of measurements shows negligible variation, indicating the high stability of the cobalt sensor.

#### 2.2.4 Effect of Interfering Ions on the Phosphate Sensor

The interfering ions (e.g., nitrate, sulfate, acetate, carbonate, and iodide) were selected because they are widely found in various environmental samples (e.g., in river water) as well as in drinking water.^47^ These ions are generally present at concentrations in the ∼10^–3^ to 10^–5^ M range.^48^ The interference of these ions on the sensor response was tested by measuring the CV of the orthophosphate (H_2_PO_4_^–^) ions (∼1 mM) in the presence of five times the concentration of the interfering ions (∼5 mM). The bar chart in Figure 2f shows the variation in the phosphate sensor current response in the presence of various interfering cations (evaluated at ∼−1.0 V cathodic peak potential). These results showed a deviation of ±20% due to the presence of interfering ions, with the highest effect being found with the presence of humic acid (±15%) in the water sample. Other ions have a moderate effect of ∼±5% on sensor performance, supporting the selectivity of the cobalt-based phosphate sensor. The shift in current response was quantified using eq 5:
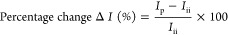
5where *I*_p_ is the phosphate current value and *I*_ii_ is the interfering ion current. Collectively, these results show that the cobalt electrode exhibits good selectivity and sensitivity for phosphate ions, strengthening the potential of the cobalt sensor for biological and agricultural applications where the environmental interferences mentioned previously are commonly observed.^27,49,50^

### 2.3 Surface Characterization

**Figure 3:**
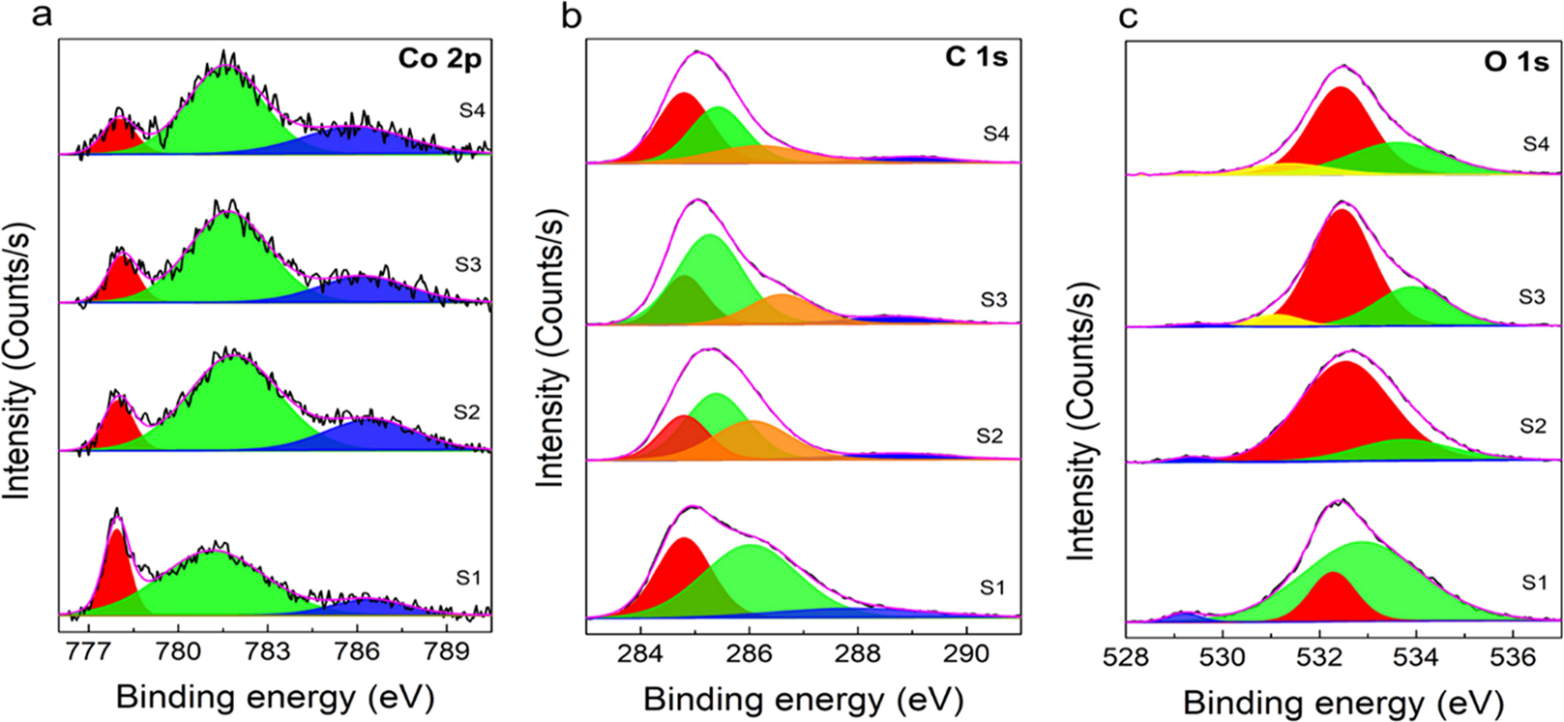
Deconvoluted XPS peaks of cobalt electrode. (a) Peaks for cobalt (Co), (b) peaks for carbon (C), and (c) peaks for oxygen (O). S1: bare cobalt wire, S2: KHP buffer (after 10 cycles of CV), S3: KHP buffer + KH_2_PO_4_ (after 10 cycles of CV), and S4: KHP buffer + KH_2_PO_4_ (after 20 cycles of CV).

To investigate the cobalt electrode surface chemistry under various conditions, we conducted X-ray photoelectron spectroscopy (XPS) studies on the sensors before and after the electrochemical experiments (Figure 3). Deconvolution of the XPS spectra for the fresh cobalt wire showed four orbital splitting peaks, specifically, a Co^0^ (Co 2p_3/2_) prominent peak at 778 eV, a CoO (Co 2p_3/2_) peak at 781 eV, and a Co 2p_1/2_ peak at 797 eV (Figure 3a (S1)). These values are in good agreement with data found in the literature for Co 2p core-level XPS spectra.^51,52^ After electrochemical experiments using different test solutions, the deconvoluted XPS spectra showed only two prominent peaks as seen in Figure 3a (S2–S4) due to the oxidation of Co^0^ to Co^2+^. In a 25 mM KHP buffer (Figure 3a (S2)), a peak shift was observed from the oxidation of Co^0^ (Co 2p_3/2_ at 778 eV) to Co^2+^(Co 2p_3/2_ at 781.5 eV). Similar results were also observed in previous studies.^29,53^ Similarly, for the cobalt electrodes exposed to phosphate ions (Figure 3a (S3)), a shift in the peak in the XPS peaks was observed due to the oxidation of Co^0^ (Co 2p_3/2_) at 778 eV to Co^2+^ (Co 2p_3/2_) at 782.5 eV due to the formation of the Co_3_(PO_4_)_2_ complex on the electrode surface. Furthermore, the appearance of the Co^2+^ (Co 2p_3/2_) peak at 783.5 eV is further evidence of the formation of the Co_3_(PO_4_)_2_ complex after additional CV cycles (Figure 3a (S4)). Peaks for carbon at 284 eV correspond to the C–C bonding and at 286 eV to the C–O adventitious carbon peaks (Figure 3b). Peaks for oxygen were observed at 532 eV, specific to C–O bonding, and H–O–C stretching was observed at 533 eV.

To validate the XPS results and support the evidence of the formation of the Co_3_(PO_4_)_2_ complex, we collected energy dispersive X-ray spectroscopy (EDAX) spectra of the cobalt electrode under the same test conditions. The results are presented in Supplementary Information Figure S4. The EDAX results showed a decrease in the weight % for Co in the *K-shell* from 98.1 to 93.7% due to the oxidation of cobalt observed in the XPS studies.

### 2.4 Surface Charge Transfer Processes on a Cobalt Electrode

**Figure 4:**
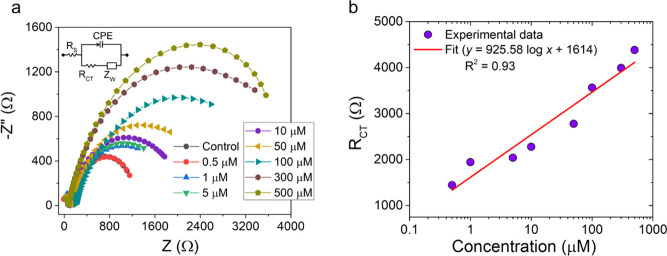
(a) Nyquist curve at different phosphate ion concentrations in the range of 0 to 500 μM. The inset shows the equivalent circuit. (b) Plot of charge transfer resistance at different phosphate ion concentrations.

The charge transfer processes occurring on the cobalt electrode surface due to the diffusion of the phosphate ions were investigated using electrochemical impedance spectroscopy (EIS). The impedimetric response of the cobalt sensor was measured as a function of the phosphate concentration in the frequency range of 1.0 Hz to 1.0 MHz with an AC amplitude of 10 mV vs Ag/AgCl. The semicircular curve in EIS, referred to as the Nyquist curve, corresponds to the electron transfer process, its diameter suggesting the electron/charge transfer resistance (*R*_CT_) that depends on the properties of the electrode–electrolyte interface.^54^ The corresponding Nyquist curves at different phosphate ion concentrations are shown in Figure 4. The inset of Figure 4a shows the equivalent circuit used to fit the impedance data. Here, *R*_S_ is the solution resistance, CPE the constant phase element, *Z*_W_ the Warburg impedance, and *R*(*z*) is the redox resistance that occurs due to the diffusion of the phosphate ions from the bulk to the cobalt electrode interface.^55^ The solution resistance (*R*_S_) for all experiments was found to be ∼100–200 Ω (Figure 4a). The EIS results show an increase in *R*_CT_ from 1 to 3.5 kΩ with an increase in phosphate concentration in the range of 0–500 μM (Figure 4b). The increase in the charge transfer resistance results because of the formation of a Co_3_(PO_4_)_2_ dielectric layer with the increase in the concentration of phosphate, a finding that agrees well with our proposed mechanism.

### 2.5 Electrochemical Phosphate Detection in Environmental Water Samples

**Figure 5:**
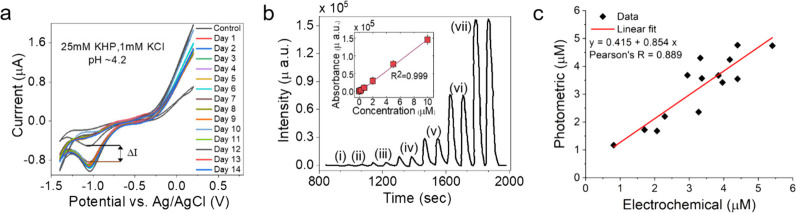
Phosphate detection in Mississippi (MS) water samples. (a) Electrochemical detection method. Cyclic voltammograms of cobalt wire as the working electrode with an Ag/AgCl reference electrode and Pt wire as a counter electrode in a 25 mM KHP buffer and a 1 mM KCl with KH_2_PO_4_; pH ∼ 4.2. (b) Photometric detection method. Absorbance recorded from a flow injection system at (i) 0, (ii) 0.1, (iii) 0.25, (iv) 0.75, (v) 2, (vi) 5, and (vii) 10 μM phosphate concentrations. Inset: Calibration curve obtained by the signals recorded from the flow injection analysis (*n* = 2). (c) Performance comparison of electrochemical versus photometric phosphate concentration.

Our CV technique was used to detect orthophosphate in water samples from the Mississippi River. This test had two objectives: (i) to demonstrate whether the calibration curve developed for the electrochemical sensor is appropriate for on-field application and (ii) to assess the interferences from the real water matrix on phosphate ion detection. The CV measurements from 14 water samples collected over a one-month period are presented in Figure 5a. The unknown phosphate concentrations in the Mississippi River water samples were determined from the calibration curve developed by using the reduction peak current (at −1.05 V). The results have also been tabulated in Supplementary Information Table S1**.** These results were validated using EPA Method 365.5.

### 2.6 Spectrophotometric Phosphate Detection in Environmental Water Samples

To validate our results from the electrochemical measurements, the EPA standard spectrophotometric method was used to determine the phosphate content in the river water samples. The phosphate signals recorded from the flow injection system from 0 to 10 μM phosphate concentrations using the spectrophotometric method can be seen in Figure 5b. The phosphate concentrations for the Mississippi River water samples determined from the calibration curve are presented in Figure 5b (inset), and the results are tabulated in Supplementary Information Table S2. The performance comparison of the electrochemical versus the photometric phosphate concentration is presented in Figure 5c. The Pearson’s *R*, determined from the statistical analysis of the data presented in Figure 5c, was found to be 0.889, indicating a good fit of the electrochemical and photometric phosphate concentration**.** These results indicate that the cobalt–phosphate sensor exhibits good performance and stability for applications related to the real water matrix.

### 2.7 Disposable Microfabricated Phosphate Sensor Chips

**Figure 6:**
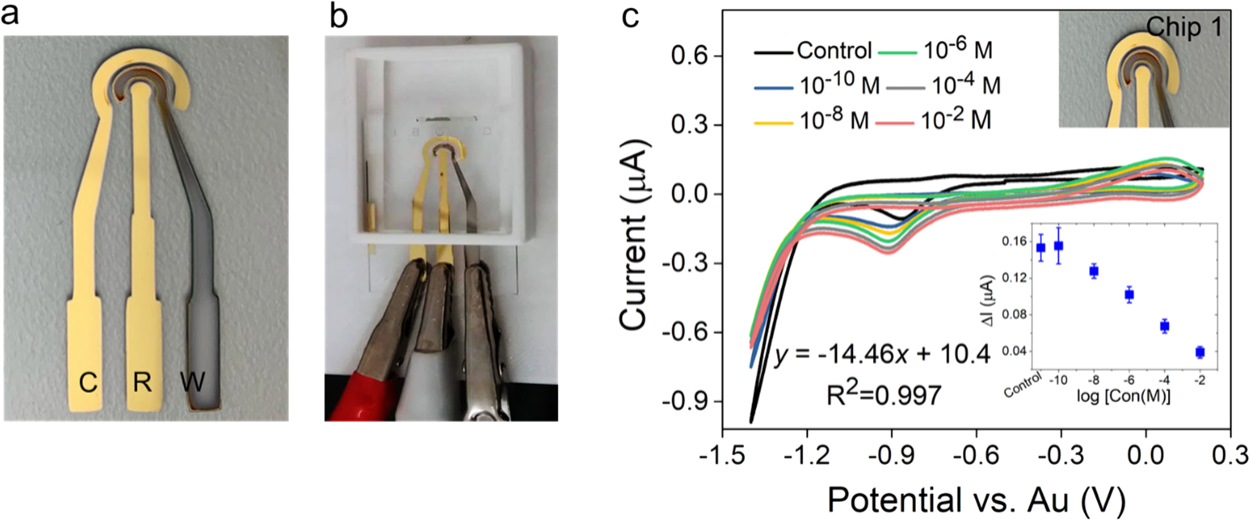
Printed electrodes on the glass substrate. (a–b) Image showing the sensor. Here, C = counter electrode, R = reference electrode, and W = working electrode. (c) Phosphate sensing using the printed electrodes. Cyclic voltammograms of Co-metal as the working electrode with Au-metal as the reference and counter electrode in a 25 mM KHP buffer and 1 mM KCl with KH_2_PO_4_ in the concentration range of 10^–10^ to 10^–2^ M at a scan rate of 50 mV/s. Inset: Phosphate sensor calibration curve showing a linear range of detection in 10^–10^ to 10^–2^ M. Note: The current signal obtained from the buffer was subtracted from all sample peak currents.

For field applications, we designed, fabricated, and tested disposable sensors on a glass substrate. Figure 6a,b show the optical images of the fabricated sensors. For simplicity and low cost, we used Au as both the reference and counter electrode, and a planar cobalt electrode was used as a working electrode. This design is also compatible with large-scale production using screen-printing technology. The electrochemical response of this fully integrated sensor was evaluated using CV testing. The CV curves in Figure 6c (and Supplementary Information Figure S8) show the phosphate selective response of these disposable sensors over dynamic concentrations ranging from 10^–10^ to 10^–2^ M. The applied potential was in the voltage range of −1.4 to 0.4 V. Due to the use of Au as the reference electrode, the cathodic dip due to the reduction of Co^2+^ to Co^0^ on our sensor shifted from the previously described position and appeared at about −1.0 V. These results are in accordance with the reduction peak observed in Figure 6c at −1.0 V with our optimized cobalt-based phosphate sensor. The anodic peak also increased with the increase in the phosphate concentrations, similar to the results observed with the bulk Co electrodes. The phosphate sensor calibration curve (provided in the inset in Figure 6c) shows a linear trend from 10^–10^ to 10^–2^ M with a detection limit of ∼10^–7^ M (Supplementary Information Table S4). The *R*^2^ coefficient of the linear fit was found to be 0.99. We have obtained an order of magnitude improved limit of detection in comparison to previously published reports using a cobalt sensor (Supplementary Information Table S5). The disposable sensor also exhibited significant reproducibility with a chip-to-chip variation of 0.03%. Additionally, Au metal deposition is easy and corrosion-resistant and does not easily oxidize, ensuring long-term stability for mass production. The performance of the glass-printed cobalt–phosphate sensor was found to be better compared to the conventional bulk Co wire. Furthermore, the printed sensor can be integrated with a point-of-application device, making this sensor chip applicable for portable on-field environmental applications.

## 3 Conclusions

Portable devices are needed for on-field applications to monitor water pollutants to help alleviate water contamination problems. We report the design, fabrication, and testing of a low-cost, portable, and sensitive electrochemical Co-based sensor for the quantitative determination of phosphate. The bulk sensor showed a linear response for phosphate ions within a dynamic range of 10^–7^ to 10^–3^ M. The limit of detection of the bulk sensor was found to be 10^–7^ M. The sensor performance was influenced by DO and pH interferences. Furthermore, humic acid exhibited the greatest effect on phosphate sensor performance. The results suggest that simultaneous measurement of DO and pH is needed for accurate estimation and successful implementation for on-field environmental application.

## 4 Experimental Section

### 4.1 Materials

Potassium phosphate (KH_2_PO_4_) and potassium hydrogen phthalate (KHP) were purchased from Ward Science (Rochester, NY, USA). Potassium chloride (KCl), sodium carbonate (Na_2_CO_3_), potassium iodide (KI), sodium nitrate (NaNO_3_), and calcium acetate (C_4_H_6_O_4_Ca) were ordered from VWR-Chemicals (Missouri, TX, USA). Potassium sulfate (K_2_SO_4_) was acquired from EMD Chemicals (Burlington, MA, USA). Cobalt wire (0.5 mm diameter, 99.995% pure) and humic acid were purchased from Alfa Aesar (Ward Hill, MA, USA). The double-distilled water obtained from Milli-Q was utilized for making solutions of all analytes.

### 4.2 Electrochemical Measurements

An electrochemical workstation (Potentiostat/Galvanostat Interface1010E, Gamry Instruments, Warminster, PA, USA) was employed to record the cyclic voltammograms (CV) and the chronoamperometry (CA) response. Initial phosphate sensing was demonstrated using a three-electrode (working, counter, and reference electrode) configuration, with a Co wire (diameter = 0.5 mm, active area ∼ 1.85 cm^2^) serving as a working electrode, Pt wire as a counter electrode, and Ag/AgCl as a reference electrode.

A scanning rate of 50 mV/s was used for all CV measurements, and the applied potential was scanned for four cycles in the voltage range of −1.4 to 0.4 V in a 25 mM KHP buffer and 1 mM KCl with KH_2_PO_4_ in a concentration range of 5 × 10^–7^ to 5 × 10^–4^ M at pH ∼ 4.2. The sensor calibration curve and the limit of detection were evaluated for these concentration ranges. The CA measurements were performed in the same concentration range while applying a step potential of 0.25 V with a timestep of 40 s. Square wave pulsed voltammetry (SQV) experiments were conducted using the same setup within a potential window from 0.4 to −1.4 V vs Ag/AgCl using a 50 mV amplitude, a potential step of 0.75 mV, and a frequency of 25 Hz.

The sensing performance of the electrodes was evaluated by conducting voltammetry experiments under various conditions including (i) the effect of dissolved oxygen, (ii) the effect of pH, and (iii) the effect of common interfering ions normally present in environmental water samples. The effect of dissolved oxygen was evaluated by recording and comparing CV measurements at the same aqueous phosphate concentration as prepared after air/O_2_ saturation through bubbling air for ∼30 min and after N_2_ saturation by bubbling high-purity N_2_ for up to ∼30 min. The concentrations of oxygen in the test solutions were monitored using a commercial oxygen electrode. The solutions post-bubbling were immediately used for conducting CV measurements under identical experimental settings. The influence of pH was evaluated using CV and SQV in the pH range from 2 to 11 by adjusting to the desired pH using dilute HCl (1 M) and/or NaOH (0.75 M). The effect of interference from common ions, specifically sulfate, carbonate, acetate, nitrate, and iodide, was recorded at different concentrations. We also evaluated the effect of natural organic matters or humic acid on the phosphate measurements.

Further electrochemical phosphate measurements were conducted using river water samples collected from the Mississippi River at Baton Rouge, Louisiana, every other day over a period of 30 days. The samples were stored at 4 °C for further analysis. From the collected river water samples, 10 mL of the water samples was filtered through a 0.2 μm syringe filter before each experiment. For the electrochemical experiments, the pH of the samples was brought to pH ∼ 4.2 by adding 25 mM KHP. Finally, the CV and CA responses were recorded as described above.

### 4.3 Characterization

An X-ray photoelectron spectroscope (XPS) from Scienta Omicron ESCA 2SR with an Al Kα X-ray source was used for cobalt electrode surface examination. The XPS spectra were obtained from an inert environment by applying 3 keV beam energy at a 45° inclination to etch the cobalt surface.

### 4.4 Spectrophotometry Measurements

For validating the electrochemical results, EPA Method 365.5 (orthophosphate in estuarine and coastal water by colorimetry) was used to determine the phosphate content in the Mississippi River water samples. An OI Analytical Flow Solutions IV auto analyzer was used for the colorimetric determination of phosphate. Briefly, the spectrophotometric determination of orthophosphate was based on the reaction of the water sample with a composite reagent of ammonium molybdate ((NH_4_)_2_MoO_4_) and antimony potassium tartarate (C_8_H_10_K_2_O_15_Sb_2_) in an acidic medium to form an antimony phosphomolybdate complex. The resulting complex reacted with ascorbic acid to produce a bluish complex, the optical absorbance of which was measured at ∼660 nm. The color intensity of the resulting sample is directly proportionate to the orthophosphate concentration.

### 4.5 Fabrication of Printed Electrodes

For the fabrication of printed electrodes (with working, counter, and reference electrodes), a design was made using AutoCAD 2016 with dimensions suitable for a standard glass slide (75 × 25 mm). This design was cut from a clear Mylar sheet using a Cameo Cutter, and the cut pieces were taped on a standard clean glass slide for metal deposition using vacuum tape. A Physical Vapor Deposition Evaporator at a base pressure of 10^–8^ Torr was used for the deposition of the metals. Briefly, a Ti layer of 35 nm was deposited at 20 Å/s (for working, counter, and reference electrodes) followed by a 200 nm Au layer deposited at 1 Å/s to create the counter and reference electrodes by masking the working electrode with a Mylar sheet. Finally, a Co layer of 300 nm was deposited at 1–5 Å/s to create the working electrode by masking the remaining areas with a Mylar sheet. The size of the sensing area was 8.9 mm^2^, and the gap between each electrode was 0.35 mm. The area of the working electrode was 2.8 mm^2^, the area of the counter electrode was 6.0 mm^2^, and the area of the reference electrode was 0.73 mm^2^. The detailed fabrication procedure with photographs is shown in the Supplementary Information.

## Supporting Information Available

Images of the electrochemical apparatus setup and the CV results for three electrodes, the chemical reactions, the Pourbaix diagram, the current–time response profile of the phosphate sensor, the EDAX spectra of cobalt electrode, the fabrication of printed electrodes, the phosphate sensing using the printed electrodes, the concentration of phosphate in the Mississippi water using the electrochemical method, the concentration of phosphate in the Mississippi water using the spectrophotometric method, the LOD calculation, and the literature on the sensing performance of phosphate sensors (PDF)
