# Supplementary material for: Printed Electrode for Measuring Phosphate in Environmental Water
Source: ACS Omega. 2021 Apr 22;6(17):11297–306. doi: 10.1021/acsomega.1c00132 (PMC8153944; doi:10.1021/acsomega.1c00132)
Supplement: Supplementary file 1 — ao1c00132_si_001.pdf [file ao1c00132_si_001.pdf]

Supplementary Information for

Printed Electrode for Measuring Phosphate in Environmental Water

Alisha Prasad[1,#], Sushant P Sahu[1,#], Sara Karoline Figueiredo Stofela[2], Ardalan Chaichi[1], Syed Mohammad Abid Hasan[1], Wokil Bam[3], Kanchan Maiti[3], Kevin M McPeak[2], Gang Logan Liu[4], Manas Ranjan Gartia[1,*]

[1]Department of Mechanical and Industrial Engineering, Louisiana State University, Baton Rouge, Louisiana, USA 70803

[2]Department of Chemical Engineering, Louisiana State University, Baton Rouge, Louisiana, USA 70803

[3]Department of Oceanography and Coastal Sciences, Louisiana State University, Baton Rouge, Louisiana, USA 70803

[4]Department of Electrical and Computer Engineering, University of Illinois, Urbana-Champaign, Illinois, USA 61801

* All correspondences should be addressed to: mgartia@lsu.edu; [#]Equal contribution

Table of Content

Figure S1 Image of electrochemical apparatus setup for Three-electrode……….…..………... S-3

Section S1 Chemical reactions …………………………….……..……………..……….…..… S-3

Figure S2 Current-time response profile of the phosphate sensor ……………......……...…... S-4

Figure S3 Pourbaix diagram ……………………………………………..……........……...… S-4

Figure S4 EDAX spectra of cobalt electrode……………..……...……………........……...… S-5

Figure S5 Effect of pH on the current response of the sensor ……………………………….. S-6

Figure S6 Fabrication of Printed Electrodes …..…………………………………………….... S-7

Figure S7 Step by step fabrication process of Printed Electrodes …..……………………….. S-7

Figure S8 Phosphate sensing using the printed electrodes……………………………………...S-8

Figure S9 Evaluation of the effect of dissolved oxygen on the phosphate sensor …..….S-8

Table S1 Concentration of Phosphate in Mississippi water by Electrochemical method…........S-9

Table S2 Comparison of electrochemical and colorimetric sensor data ……………………….S-9

Table S3 Concentration of Phosphate in Mississippi water by Spectrophotometric method.....S-10

Table S4: LOD Calculation…..S-10

Table S5: Literature on sensing performance of phosphate sensor…..S-11

Table S6: Evaluation of cobalt sensor response in presence of Dissolved Oxygen …..............S-11

References ………………………………………………………………………………….S-12

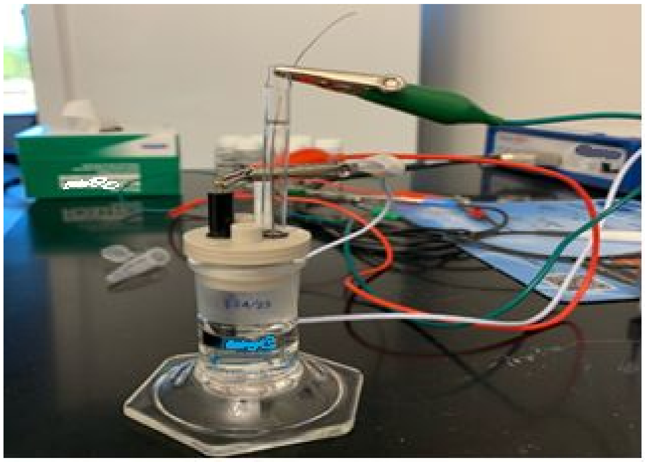

Figure S1. Electrochemical apparatus setup. Green: Working Electrode; Red: Counter Electrode; White: Reference Electrode

Section. S1 Chemical reactions

$2Co + 2H_2O \Leftrightarrow 2CoO + 4H^+ + 4e^-$……………………...……………...…...…...…... (1)

$O_2 + 4H^+ + 4e^- \Leftrightarrow 2H_2O$………………………………………………...…….…… (2)

$2Co + O_2 \Leftrightarrow 2CoO$…………………………………………………….........(3)

$3CoO + 2H_2PO_4^- + 2H^+ \Leftrightarrow Co_3(PO_4)_2 + 3H_2O$ at pH 4.0…...……………………….....…...… (4)

$3CoO + 2HPO_4^{2-} + H_2O \Leftrightarrow Co_3(PO_4)_2 + 4OH^-$ at pH 8.0…………………………….....…. (5)

$3CoO + 2PO_4^{3-} + 3H_2O \Leftrightarrow Co_3(PO_4)_2 + 6OH^-$ at pH 11.0…………………………...….......(6)

The chemical reactions from equation (1) to (6) are explained below:

1. Reduction (cathode): The phosphate ions associate at the Co electrode surface, generating Co^{2+} to Co^0 (cathodic peak) redox couple.
2. Oxidation (anode): The Co^{2+} /Co^0 redox couple influences the electrode surface and leads to formation of $Co_3(PO_4)_2$ (anodic peak).
3. Acidic state (pH:4.0): Corresponds to dihydrogen phosphate ($H_2PO_4^-$) (Ref. 1).
4. Neutral state (pH:8.0): Corresponds to hydrogen phosphate (HPO_4^{2-}) (Ref. 2).
5. Basic state (pH:11.0): Corresponds to phosphate (PO_4^{3-}) ions (Ref. 2).

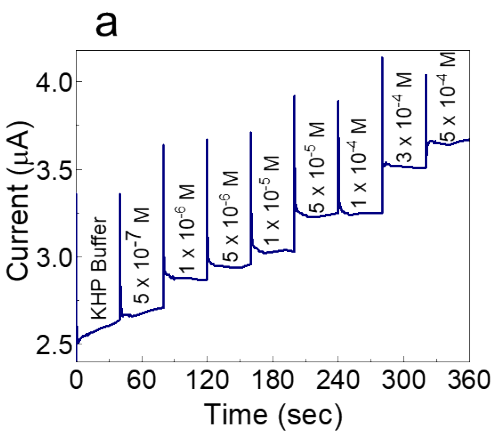

Figure S2. Current-time response profile of the phosphate sensor (a) Chronoamperograms showing the phosphate sensor response at different phosphate concentrations. (b) Phosphate sensor calibration curve showing linear range of detection from 10^{-7} M to 10^{-4} M with bulk cobalt wire.

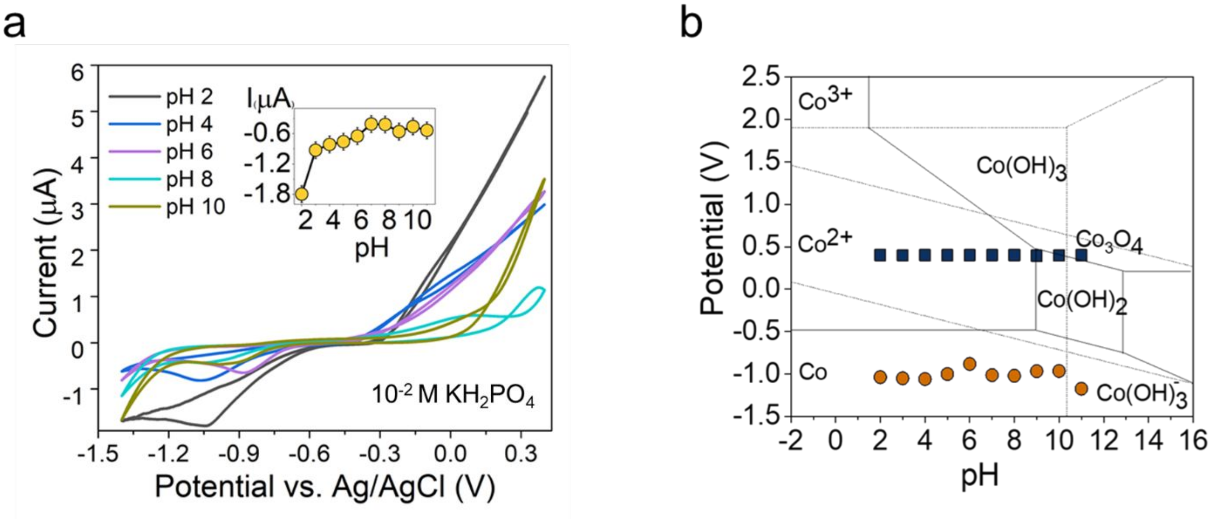

Figure S3. (a) Effect of pH on the CV. (b) Pourbaix diagram of cobalt sensor showing thermodynamic pH/potential characteristics in the pH range of 2 to 11. The pre-catalytic redox features evident in the CVs (**Figure S3a**) is denoted by symbol ■ at the anode and ● at the cathode, respectively.

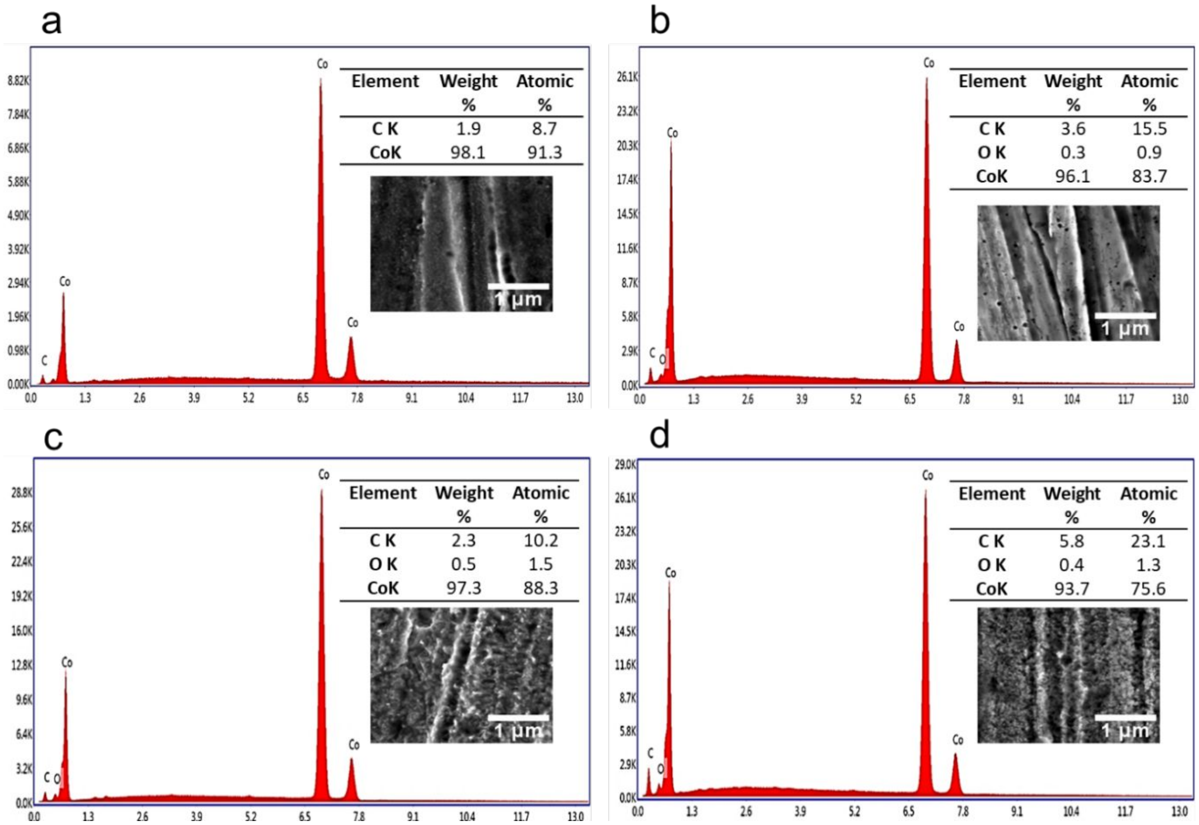

Figure S4. EDAX spectra of cobalt electrode (a) Bare Cobalt wire, (b) in KHP Buffer (10 cycles CV), (c) in KHP Buffer + KH_2PO_4 (10 cycles CV), and (d) in KHP Buffer + KH_2PO_4 (20 cycles CV). From the EDAX spectra of the cobalt electrode presented in **Figure S4** under various test conditions a decrease in the EDAX weight % across test solutions (a-d) from 98.1% to 93.7 % for element Co in the *K-shell* was observed which is due to the oxidation of cobalt (Co^0 to Co^{2+}) as explained in the XPS studies.

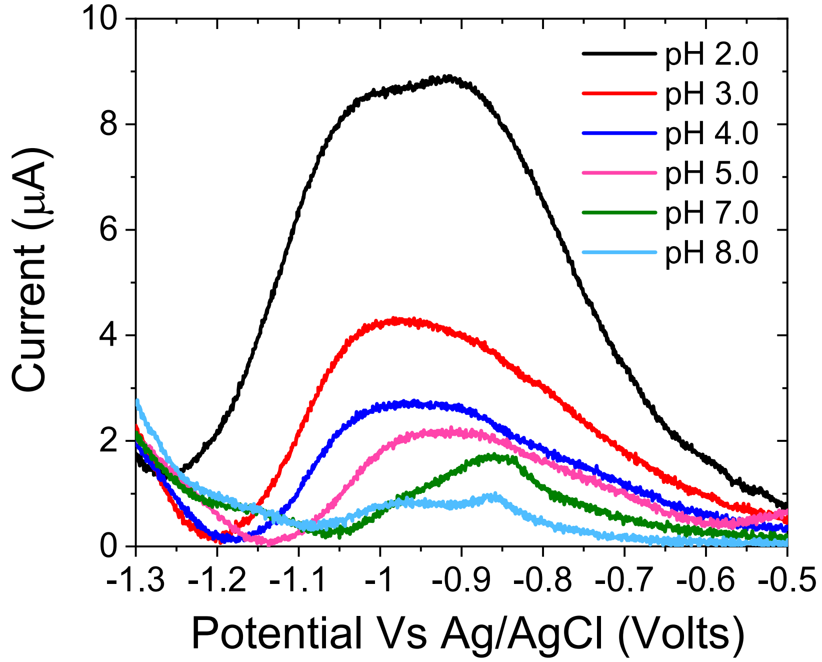

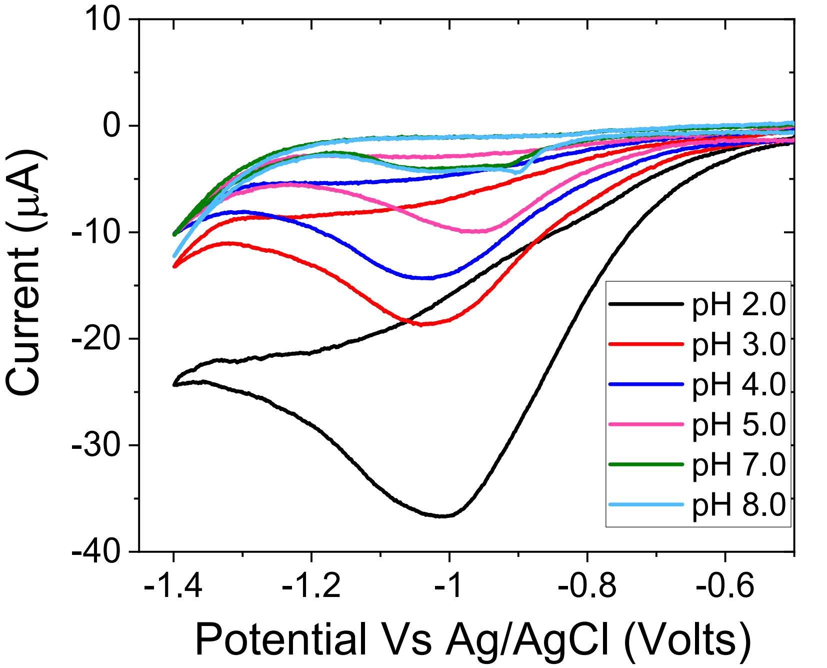

Figure S5. (Top) Square wave pulsed voltammograms showing the characteristic cathodic current response of sensor, and (Bottom) Cyclic voltammograms showing the characteristic cathodic current response of sensor measured for 100 μM phosphate in the pH range 2 to 8.

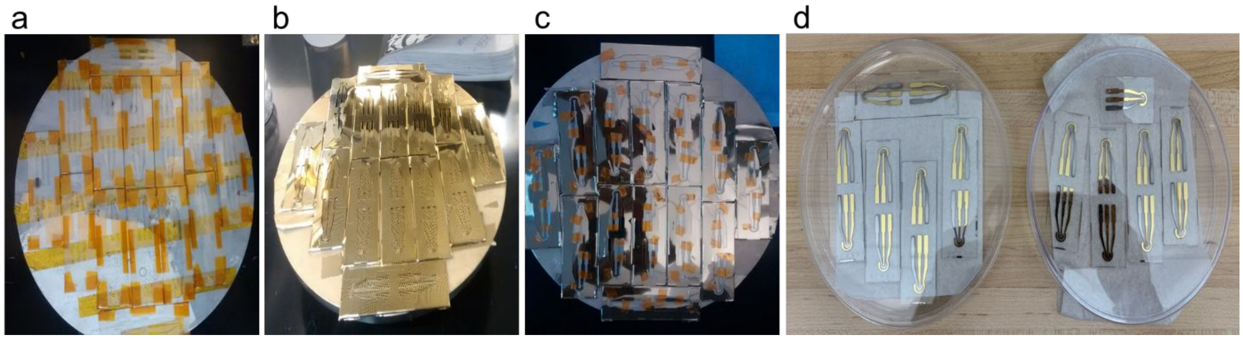

Figure S6. Fabrication of Printed Electrodes. (a) Loading Mylar pieces taped on glass slide for metal deposition (b) After deposition of Gold layer (c) After deposition of Cobalt layer (d) Mass Production of Printed electrodes

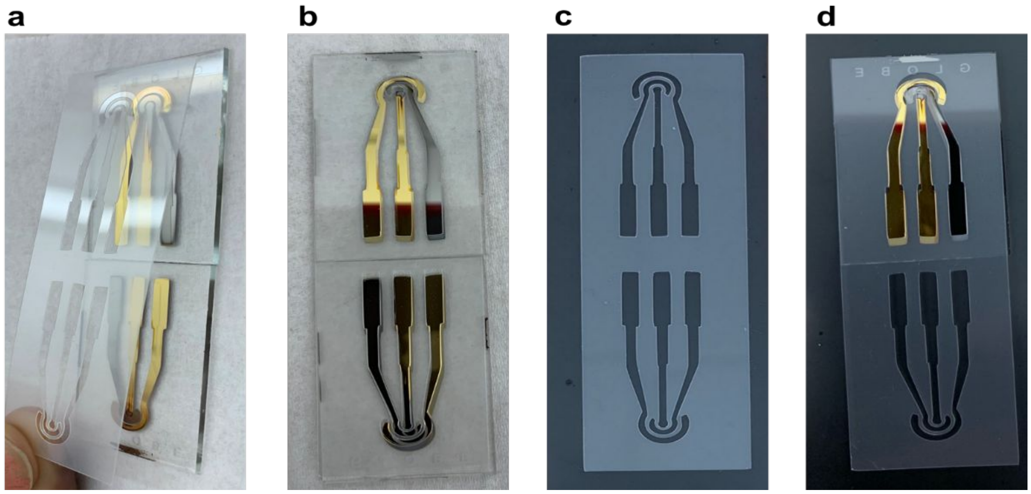

Figure S7. Step-by-step process of making glass printed electrodes. (a) Masking using Mylar sheets (b) Final output after metal deposition (c-d) In black background.

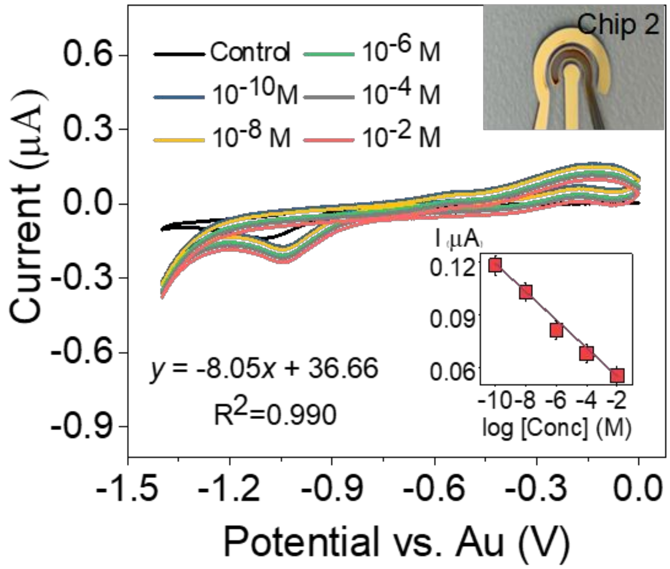

Figure S8. Phosphate sensing using the printed electrodes. Cyclic voltammograms of Co-metal as working electrode with Au-metal as reference electrode and counter electrode in 25 mM KHP buffer and 1mM KCl with KH_2PO_4 in the concentration range of 10^{-10} M to 10^{-2} M with a scan rate of 50 mV s^{-1}. Inset: Phosphate sensor calibration curve showing linear range of detection in 10^{-10} M to 10^{-2} M. Note: The current signal obtained from the buffer was subtracted from all sample peak currents.

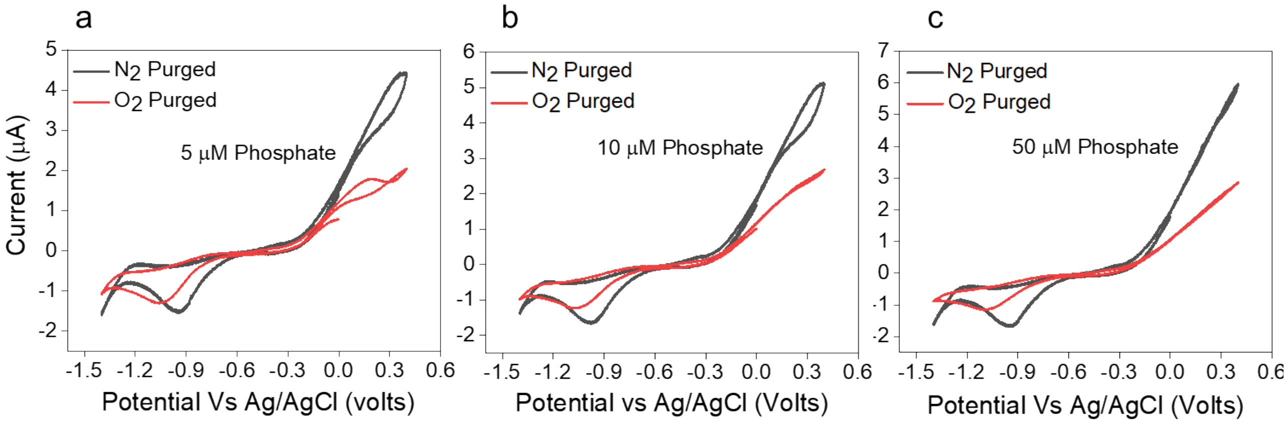

Figure S9. Evaluation of the effect of dissolved oxygen on the phosphate sensor. (a) 5µM (b) 10 µM (c) 50µM of orthophosphate.

Table S1: Concentration of Phosphate in Mississippi water samples by Electrochemical method

DATE	Phosphate Concentration (μM)		Mean (μM)	Mean (mg/L)	Standard Deviation	Coefficient of Variation (CV%)
1	5.66	5.4	5.53	0.536	0.18	3.3
2	4.27	5.1	4.69	0.454	0.59	12.5
3	4.51	4.57	4.54	0.440	0.04	0.9
4	4.77	4.25	4.51	0.437	0.37	8.1
5	4.34	3.9	4.12	0.399	0.31	7.5
6	1.65	1.8	1.73	0.167	0.11	6.1
7	4.94	5.77	5.36	0.519	0.59	10.9
8	4.83	4.49	4.66	0.452	0.24	5.2
9	3.93	3.52	3.73	0.361	0.29	7.8
10	2.94	2.38	2.66	0.258	0.40	14.9
11	3.01	3.34	3.18	0.308	0.23	7.4
12	1.01	1.2	1.11	0.107	0.13	12.2
13	2.64	2.31	2.48	0.240	0.23	9.4
14	1.37	1.21	1.29	0.125	0.11	8.8

Table S2: Comparison of electrochemical and colorimetric sensor data

Days	Electrochemical	Colorimetric	Difference	Difference (Abs)
1	5.53	4.74	0.79	0.79
2	4.69	4.76	-0.07	0.07
3	4.54	4.3	0.24	0.24
4	4.51	3.55	0.96	0.96
5	4.12	3.42	0.7	0.7
6	1.73	1.72	0.01	0.01
7	5.36	4.24	1.12	1.12
8	4.66	3.67	0.99	0.99
9	3.73	3.57	0.16	0.16
10	2.66	3.68	-1.02	1.02
11	3.18	2.36	0.82	0.82
12	1.11	1.17	-0.06	0.06
13	2.48	2.2	0.28	0.28
14	1.29	1.68	-0.39	0.39

Table S3: Concentration of Phosphate in Mississippi water samples by Spectrophotometric method

DATE	Phosphate (μM)
**Day 1**	4.74
**Day 2**	4.76
**Day 3**	4.3
**Day 4**	3.55
**Day 5**	3.42
**Day 6**	1.72
**Day 7**	4.24
**Day 8**	3.67
**Day 9**	3.57
**Day 10**	3.68
**Day 11**	2.36
**Day 12**	1.17
**Day 13**	2.20
**Day 14**	1.68

Table S4: LOD Calculation

Iteration	Cyclic Voltammetry Buffer Current	
	**Bulk (mA)**	**Printed Chip (mA)**
**1**	-0.001307441	-0.000115847
**2**	-0.001307032	-0.000115689
**3**	-0.001307437	-0.000116001
**STDEV**	2.35E-07	1.56351E-07
**LOD**	7.05E-07	4.69052E-07

Table S5: Analytical parameters and comparison of potentiometric sensing performance of phosphate sensor with previous published reports

Sl. No.	Active material (Ion Selective Electrode)	Dynamic linear range	Detection limit	References	Year
**1**	Glass Printed Cobalt electrode	10^{-10} to 10^{-2} M	10^{-7} M	Present Work	
**2**	Screen Printed electrode modified with carbon black nanoparticles	$10x10^{-6}$ to $80x10^{-6}$ M	$6x10^{-6}$ M	3	2015
**3**	Cobalt microelectrode	10^{-5} to 10^{-1} M	$10^{-5.1}$ M	4	2009
**4**	Planar Cobalt microelectrode	5×10^{-5} to 5×10^{-2} M	10^{-5} M	5	2006
**5**	Cobalt wire	10^{-4} to 10^{-2} M	Not mentioned	2	1996

Table S6: Evaluation of electrochemical phosphate sensor response in presence of Dissolved Oxygen

**Phosphate Concentration (µM)**	5	10	50
**I (N_2) (mA) (At -1.05 V)**	-0.00153	-0.00164	-0.00166
**I (O_2) (mA) (At -1.05 V)**	-0.00129	-0.00121	-0.00116
**Percentage Difference (%) [(N_2 - O_2)/N_2 *100]**	15.68	26.21	30.12
**Correlation Coefficient (%) [$I_{(N2 - O2)}$ / $DO_{(N2 - O2)}$ *100]**	0.004	0.007	0.008

References

(1) Xiao, D.; Yuan, H.-Y.; Li, J.; Yu, R.-Q., Surface-modified cobalt-based sensor as a phosphate-sensitive electrode. *Anal. Chem.* **1995,** *67* (2), 288-291.
(2) Meruva, R. K.; Meyerhoff, M. E., Mixed potential response mechanism of cobalt electrodes toward inorganic phosphate. *Anal. Chem.* **1996,** *68* (13), 2022-2026.
(3) Talarico, D.; Cinti, S.; Arduini, F.; Amine, A.; Moscone, D.; Palleschi, G., Phosphate detection through a cost-effective carbon black nanoparticle-modified screen-printed electrode embedded in a continuous flow system. *Environ. Sci. Technol.* **2015,** *49* (13), 7934-7939.
(4) Lee, W. H.; Seo, Y.; Bishop, P. L., Characteristics of a cobalt-based phosphate microelectrode for in situ monitoring of phosphate and its biological application. *Sens. Actuators B: Chem.* **2009,** *137* (1), 121-128.
(5) Zou, Z.; Han, J.; Jang, A.; Bishop, P. L.; Ahn, C. H., A disposable on-chip phosphate sensor with planar cobalt microelectrodes on polymer substrate. *Biosens. Bioelectron.* **2007,** *22* (9-10), 1902-1907.
